# 

**Published:** 1863-10

**Authors:** 


					﻿FOR SALE.
We desire to direct attention to the card in the present num-
ber of the Register, advertising the Toland dental depot for sale.
This establishment has a good basis for a large and profitable
dental furnishing business, and if well managed must make
money. We hope some one will take hold of it, who will give
it his personal attention, and press it along with energy. T.
A. JONES’ DENTAL DEPOT,
710 BROADWAY, NEW YORK.
-------------
The co-partnership heretofore existing under the firm of JONES
& WHITE, is dissolved, to take effect May 1st, 1861. All debts
due the firm at the New York House, to that date, together with
all charges for Gold and Silver Plate, and Jones & White’s Gold
Foil to this date, will be settled at my office 710 JBroadway, New
York, where I shall keep conslantly on hand a large and well se-
lected stock of TEETH, of Messrs. Orum & Armstrong’s manu-
facture, which are pronounced by many first class dentists a supe-
rior article both for strength and beauty. Three first premiums
'awarded for Artificial Teeth, also a good assortment of D. H.
Porter’s Teeth. Also, an entire stock of Dental Instruments, ot
the very best manufactures, together with every article used in the
profession. The usual discount made on cash bills cf 20 dollars
and upwards
1	Y-x o
				

